# The role of PRP in the healing of disc degeneration and the effect of local anesthetics on PRP

**DOI:** 10.3389/fbioe.2025.1613148

**Published:** 2025-06-18

**Authors:** Ahmet Mert, Ayse Ikinci Keles, Murat Aydin, Huseyin Serkan Erol, Osman Fikret Sonmez

**Affiliations:** ^1^ Department of Orthopedics and Traumatology, Private Pendik Yüzyıl Hospital, Istanbul, Türkiye; ^2^ Department of Histology and Embryology, Aksaray University, Faculty of Medicine, Aksaray, Türkiye; ^3^ Department of Orthopedics and Traumatology, Private Buyuk Anadolu Hospital, Istanbul, Türkiye; ^4^ Department of Biochemistry, Faculty of Veterinary Medicine, Kastamonu University, Kastamonu, Türkiye; ^5^ Department of Neurosurgery, Tepecik Training and Research Hospital, Izmir, Türkiye

**Keywords:** inflammation, intervertebral disc degeneration, local anesthesia, platelet-rich plasma, rat

## Abstract

**Aim:**

This study aimed to investigate the regenerative effects of PRP on an experimental rat model of disc degeneration using histological and biochemical parameters. Additionally, we evaluated whether ropivacaine, a local anesthetic commonly used in clinical practice, affects the efficacy of PRP.

**Methods:**

Rats were randomly divided into five groups as control and treatment groups. Disc degeneration models were established using appropriate procedures. On the intervention day, PRP was prepared from whole blood collected from the rats. PRP, PRP + ropivacaine, or ropivacaine alone was administered at the appropriate doses and according to standardized protocols.

**Results:**

In the untreated disc degeneration groups, annulus fibrosus (AF) and nucleus pulposus (NP) boundaries were indistinct, disc morphology was disrupted, collagen structures in the AF were degenerated or irregular, and vacuolization, interstitial edema, and necrotic tissue remnants were observed in the NP region. In contrast, in groups treated with PRP and PRP + ropivacaine, a reduction in edema and vacuolization, disappearance of necrotic tissue, restoration of distinct NP and AF boundaries, and decreased atrophy and cellular clustering in NP cells were observed. Biochemical analysis showed that IL-6 and TNF-α levels were within normal ranges in the groups treated with PRP and PRP + ropivacaine, whereas these levels remained elevated in the untreated disc degeneration groups, indicating ongoing effects of degeneration.

**Conclusion:**

This study demonstrates the regenerative effects of PRP in disc degeneration through histological and biochemical parameters. Furthermore, the addition of ropivacaine to PRP did not exert any negative effects on PRP’s regenerative properties.

## Introduction

Platelet-rich plasma (PRP) is obtained by centrifuging whole blood at a specific speed and duration to separate the platelet- and fibrin-rich fractions from other blood components (Dashore et al., 2021). The growth factors contained in PRP are expected to exert a healing effect by triggering inflammation. In recent years, PRP has found widespread use in clinical practice, particularly in orthopedics (Garcia et al., 2020). It has become a widely preferred treatment method with significant success in managing conditions such as osteoarthritis, ligament injuries, lateral epicondylitis, disc degeneration, calcaneal spur, nonunion fractures, rotator cuff pathologies, and bursitis (Mlynarek et al., 2016).

The frequent use of PRP in clinical practice has led to ongoing discussions regarding its application techniques and preferred administration methods. One major concern is the intense pain experienced at the injection site during administration and in the surrounding area post-procedure, which can impact patient compliance and willingness to initiate treatment. Due to this severe pain, patients often fail to complete multi-dose treatment protocols, such as two- or three-dose regimens, leading to suboptimal or unsuccessful outcomes. To enhance patient comfort during and after PRP administration, some clinicians prefer combining PRP with a local anesthetic, while others avoid this approach due to concerns that local anesthetics may impair PRP’s therapeutic effects.

A review of the literature reveals that there is insufficient scientific evidence to determine whether local anesthetics interfere with PRP efficacy (Dregalla et al., 2021; D’souza et al., 2024). In this animal study, we aimed to evaluate the potential impact of a commonly used local anesthetic on PRP effectiveness, while also assessing the therapeutic role of PRP in disc degeneration using histological and biochemical parameters.

## Materials and methods

### Animals and experimental procedures

This study was approved by the Niğde Ömer Halisdemir University Ethical Committee, Türkiye (approval date: 26 September 2022, protocol no: 2022/11), and conducted according to the principles outlined in the *Guide for the Care and Use of Laboratory Animals* (NIH Publication No. 85-23, revised 1996).

Thirty male Sprague-Dawley rats (10–12 weeks old) were randomly assigned to five groups.

1. Sham + Local Anesthesia Group (S + LAG) (n = 6): No surgical procedure was performed on rats. A single dose of local anesthetic (0.025 mL) was administered via epidural (ED) injection at the Co8-9 level.
2. Sham + Platelet-Rich Plasma Group (S + PRPG) (n = 6): No surgical procedure was performed on rats. A single dose of PRP (0.1 mL) was administered via ED injection at the Co8-9 level (Gullung et al., 2011).
3. Intervertebral Disc Degeneration Group (IVDDG) (n = 6): Rats underwent intervertebral disc degeneration without any additional treatment.
4. Intervertebral Disc Degeneration + Platelet-Rich Plasma Group (IVDDG + PRPG) (n = 6): Rats in the IVDDG group received a single dose of PRP (0.1 mL) via ED injection at the Co8-9 level.
5. Intervertebral Disc Degeneration + Platelet-Rich Plasma + Local Anesthesia Group (IVDDG + PRPG + LAG): Rats in the IVDDG group received a single dose of PRP (0.1 mL) combined with local anesthetic (0.025 mL) via ED injection at the Co8-9 level.

Although some studies have reported the chondrotoxic and discotoxic effects of local anesthetic agents, a study by Cai et al. suggested that ropivacaine exhibits minimal toxicity and can be used when necessary (Cai et al., 2014). Based on this evidence, we administered 2 mg/mL ropivacaine at a dose of 0.025 mL (Naropin 2 mg/mL, 10 mL (5 ampules); AstraZeneca, Cambridge, England, United Kingdom).

### Preparation of platelet-rich plasma

Rats were anesthetized using a combination of ketamine (100 mg/kg) and xylazine (10 mg/kg). From each rat, 2 mL of blood was drawn via the tail vein using a 21-gauge needle and immediately transferred to a centrifuge tube containing 0.1 mL of 0.1 M sodium citrate. The blood was then centrifuged at 500 *g* for 10 min, yielding three distinct layers. The top layer was collected and centrifuged again at 2200 × g for an additional 10 min. The upper portion of the resulting solution was isolated and used as PRP treatment (Zhang et al., 2017).

### Induction of intervertebral disc degeneration

Prior to degeneration, rats were anesthetized with ketamine hydrochloride (100 mg/kg) and xylazine hydrochloride (10 mg/kg) via intraperitoneal injection. A 4 cm dorsal midline incision was made over the caudal vertebrae (Co6-Co10), identified by tail palpation to expose the NP and AF. The Co8-9 intervertebral disc space was identified as the target site. A 20-gauge needle was inserted into the disc, rotated 360°, and held in position for 30 s to induce degeneration. The incision was then closed using non-absorbable 4–0 sutures (Issy et al., 2013; Sönmez and Keleş, 2023). On the day of the procedure, a single dose of 0.1 mL PRP and 0.025 mL ropivacaine was administered via the ED route. Body weights of all rats were recorded at the beginning and end of the experiment using a precision scale (KERN and SOHN GmbH, Balingen, Germany).

### Biochemical analysis

Serum levels of tumor necrosis factor-alpha (TNF-α) and interleukin-6 (IL-6) were measured using commercial ELISA kits (TNF-α, cat. no: ELK1396 and IL-6, cat. no: ELK1158; ELK Biotechnology CO., Ltd., Denver, CO, United States). Standard calibration curves were generated based on the reference standards provided in the kits, and results were expressed in pg/mL.

### Histological analysis

Based on previous studies indicating that disc degeneration develops within one to 4 weeks (Issy et al., 2013), all animals were sacrificed in the fifth week via cervical dislocation. Intervertebral disc tissues from the Co8-9 level were harvested, fixed overnight in 10% formaldehyde, and subsequently decalcified (Boncroft, 1996). The samples underwent routine histological processing and were stained with hematoxylin and eosin (H&E) and Verhoeff-Van Gieson (VVG) stains. Five-micron sections were obtained using a Shandon Finesse 325 microtome (Thermo Fisher Scientific Inc., Altrincham, Cheshire, United Kingdom) and examined under an Olympus BX53 microscope with the DP80 digital camera and the cellSens software v.1.17 (Olympus Corp., Tokyo, Japan). A histologist performed a blinded histopathological evaluation using coded slides (Keleş, 2020; Keleş and Süt, 2021; Keleş et al., 2022).

Degenerative changes in the AF were classified using VVG staining as follows: (0) normal disc appearance (Dashore et al., 2021); mildly serpentine with rupture (Garcia et al., 2020); moderately serpentine with rupture (Mlynarek et al., 2016); severely serpentine with mildly reversed contour (Dregalla et al., 2021); severely reversed contour; and (D’souza et al., 2024) indistinct structure (Nishimura and Mochida, 1998). H&E-stained disc tissues were assessed for NP morphology, AF morphology (lamellar organization, NP-AF border integrity, presence of tears/fissures), and NP cellularity. Histopathological scoring was conducted based on a scale of absent (0), minimal (+), moderate (++), or severe (+++) findings in ten different areas (Han et al., 2008).

### Statistical analysis

All statistical analyses were performed using SPSS software version 22.0 (IBM Corp., Armonk, NY, United States). Comparisons between groups were conducted using two-way ANOVA followed by Tukey’s *post hoc* test. Results were presented as mean ± standard deviation (SD), with p-values <0.05 considered statistically significant.

## Results

### Body weight results

No significant differences were found in body weights among the groups before the experiment, specifically between the S + PRPG and S + LAG groups (p = 0.770) as well as between the S + PRPG group and the IVDDG (p = 0.799), IVDDG + PRPG (p = 0.842), and IVDDG + PRPG + LAG groups (p = 0.958). At the end of the experiment, statistical comparisons showed no significant differences between the S + PRPG and S + LAG groups (p = 0.474), or between the S + PRPG group and the IVDDG (p = 0.713), IVDDG + PRPG (p = 0.884), and IVDDG + PRPG + LAG groups (p = 0.879) (Table 1).

**TABLE 1:** Body weights of the rats before and after the experiment.

Groups	Body weight (g)	
	Before the experiment	After the experiment
S + LAG	278.3 ± 9.31	346.7 ± 8.82
S + PRPG	279.5 ± 8.41	345.0 ± 4.86
IVDDG	279.8 ± 8.11	345.7 ± 6.86
IVDDG + PRPG	279.3 ± 7.97	346.0 ± 6.69
IVDDG + PRPG + LAG	278.7 ± 7.86	345.8 ± 8.95

S + LAG: sham + local anesthesia, S + PRPG: sham + platelet-rich plasma, IVDDG: intervertebral disc degeneration, IVDDG + PRPG: intervertebral disc degeneration + platelet-rich plasma, IVDDG + PRPG + LAG: intervertebral disc degeneration + platelet-rich plasma + local anesthesia. Data are presented as mean ± standard deviation.

### Serum IL-6 and TNF-α results

**FIGURE 1:**
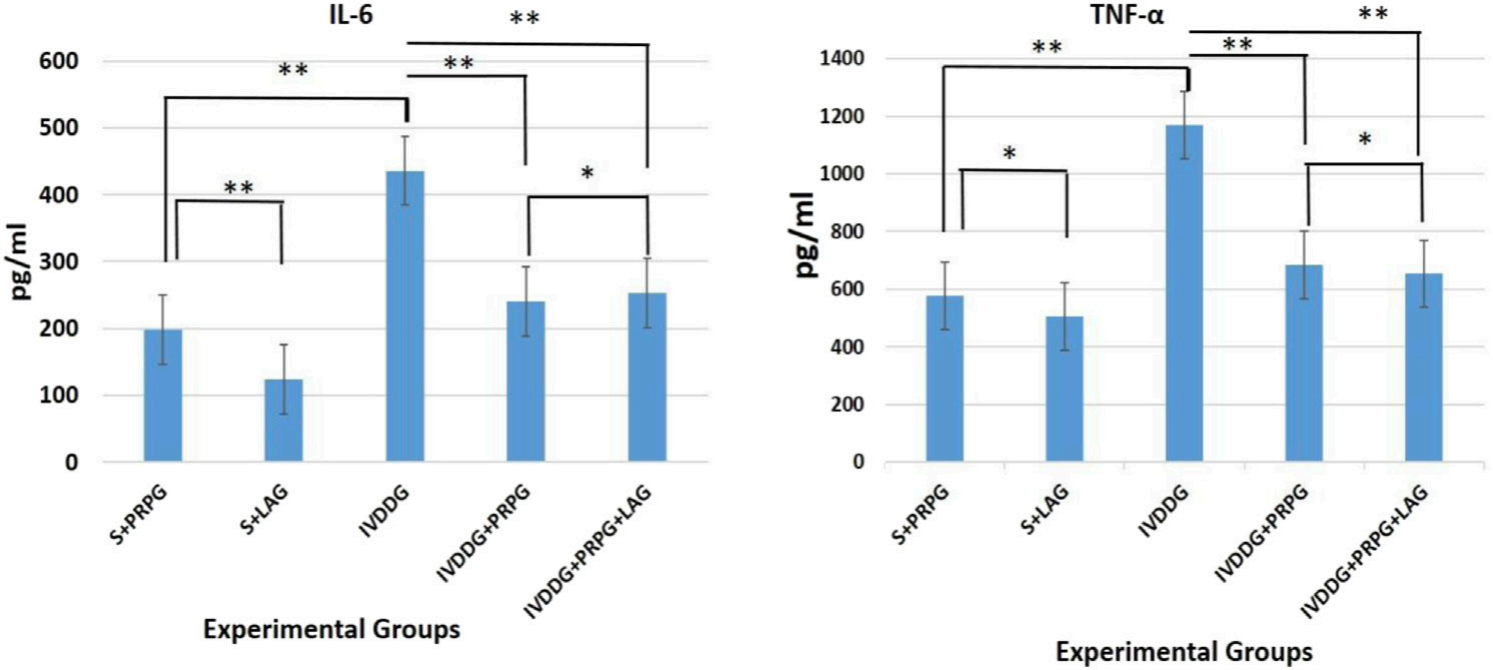
Pro-inflammatory cytokine levels in the sham + local anesthesia (S + LAG), sham + platelet-rich plasma (S + PRPG), intervertebral disc degeneration (IVDDG), intervertebral disc degeneration + platelet-rich plasma (IVDDG + PRPG), and intervertebral disc degeneration + platelet-rich plasma + local anesthesia (IVDDG + PRPG + LAG) groups. IL, interleukin; TNF, tumor necrosis factor. **p < 0.05 statistically significant and *p > 0.05 not statistically significant. The values are presented as mean ± SD.

Pro-inflammatory cytokine levels, specifically TNF-α and IL-6, were detected in serum samples from all experimental groups (Figure 1). Significant increases in IL-6 (p = 0.006) and TNF-α (p = 0.046) levels were observed in the IVDDG group compared to the S + PRPG group. When S + LAG IL-6 level was statistically increased compared to S + PRPG group(p = 0.039), while no statistical difference was found in TNF-α levels (p = 0.349). There were no statistically significant differences in IL-6 levels when comparing the S + PRP group with the IVDDG + PRPG (p = 0.052) and IVDDG + PRPG + LAG (p = 0.282) groups. Similarly, TNF-α levels did not show significant differences between the S + PRP and IVDDG + PRPG (p = 0.211) or IVDDG + PRPG + LAG (p = 0.314) groups.

Compared to the IVDDG and S + LAG groups, the treatment groups showed statistically significant reductions in IL-6 and TNF-α levels (IL-6: IVDDG + PRPG [p = 0.009] and IVDDG + PRPG + LAG [p = 0.008]; TNF-α: IVDDG + PRPG [p = 0.004] and IVDDG + PRPG + LAG [p = 0.001]).

### Histopathological results

After H&E staining, intervertebral disc tissues from all groups were evaluated. The assessments indicated that the discs and NP cells in the S + LAG and S + PRPG groups appeared normal. In the IVDDG group, the boundaries between the AF and NP were indistinct, disc shapes were ambiguous, collagen structures in the AF were degenerated or irregular, and the presence of vacuolization, interstitial edema, and necrotic tissue remnants were noted in the NP area. Additionally, NP cells exhibited atrophy and significant clustering (defined as clusters of more than 5 cells).

**FIGURE 2:**
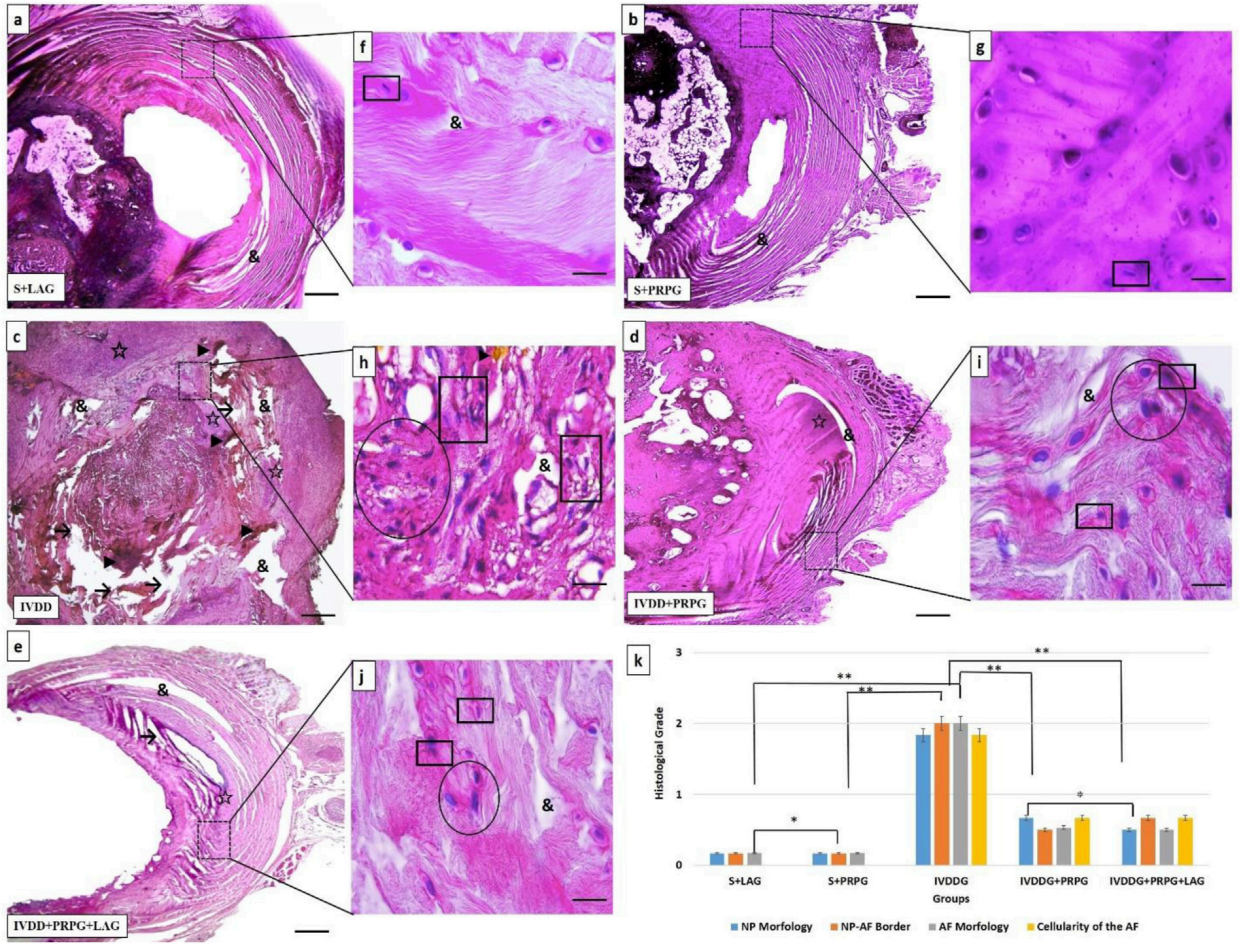
Representative light microscope images of hematoxylin-eosin-stained intervertebral disc tissue sections. The intervertebral disc tissues and nucleus pulposus (NP) cells in the sham + local anesthesia (S + LAG) and sham + platelet-rich plasma (S + PRPG) groups appeared normal. In the intervertebral disc degeneration (IVDDG) group, the borders of the annulus fibrosus (AF) and NP were unclear, with disrupted AF collagen layers. Vacuoles, interstitial edema, and necrotic tissues were observed in the disc, along with atrophy and clustering in the NP cells. In the treatment groups, IVDDG + PRPG and IVDDG + PRPG + LAG, a small amount of edema and vacuoles were detected in the intervertebral disc tissue. Additionally, a decrease in atrophic cells and cell clusters (two or 3 cell clusters) in the NP area was observed. Markings include: (&) vacuoles, (→) tissue integrity deterioration, (☆) interstitial edema, (►) necrotic tissue and degeneration, (circle shape) clusters in cells, (square shape) atrophic cells **(k)** The histological grades of different groups are presented as mean ± SD. **p < 0.05 statistically significant and *p > 0.05 not statistically significant. **(a–e)** Low magnification (×4, scale bar = 200 µm) and **(f–j)** high magnification (oil-immersion, ×100, scale bar = 10 µm).

In the treatment groups (IVDDG + PRPG and IVDDG + PRPG + LAG), reductions in edema and vacuolization were observed, along with the disappearance of necrotic tissue and clearer NP and AF boundaries. Atrophy and clustering of NP cells decreased, with occasional double or triple clusters noted. No significant histological differences were found between the IVDDG + PRPG and IVDDG + PRPG + LAG treatment groups (Figure 2).

Statistical comparisons of histological classifications revealed no significant difference between the S + LAG and S + PRPG groups (p = 0.374). However, statistically significant differences were found when comparing the sham groups (S + LAG and S + PRPG) with the IVDDG group (p < 0.001). While statistical differences were noted between the sham groups and the IVDDG + PRPG (p = 0.001) and IVDDG + PRPG + LAG (p = 0.002) groups, the average values of pathological findings decreased, indicating a regression of degeneration. Statistical comparisons of histological classifications revealed no significant difference between the IVDDG + PRPG and IVDDG + PRPG + LAG groups (p = 0.914). Statistically significant reductions were observed in the IVDDG groups compared to the treatment groups (p < 0.001) (Table 2; Figure 2).

**TABLE 2:** Averages of histological classification of intervertebral disc tissues stained with hematoxylin and eosin.

Groups	NP morphology	NP-AF Border	AF morphology	Cellularity of the NP
S + LAG	0.17 ± 0.41	0.17 ± 0.41	0.17 ± 0.41	0.00 ± 0.00
S + PRPG	0.17 ± 0.41	0.17 ± 0.41	0.17 ± 0.41	0.00 ± 0.00
IVDDG	1.82 ± 0.41	2.00 ± 0.00	2.00 ± 0.00	1.82 ± 0.41
IVDDG + PRPG	0.66 ± 0.55	0.5 ± 0.52	0.66 ± 0.55	0.67 ± 0.52
IVDDG + PRPG + LAG	0.50 ± 0.55	0.67 ± 0.52	0.50 ± 0.55	0.5 ± 0.52

NP: nucleus pulposus, AF: annulus fibrosus, S + LAG: sham + local anesthesia, S + PRPG: sham + platelet-rich plasma, IVDDG: intervertebral disc degeneration, IVDDG + PRPG: intervertebral disc degeneration + platelet-rich plasma, IVDDG + PRPG + LAG: intervertebral disc degeneration + platelet-rich plasma + local anesthesia. Data are presented as mean ± standard deviation.

**FIGURE 3:**
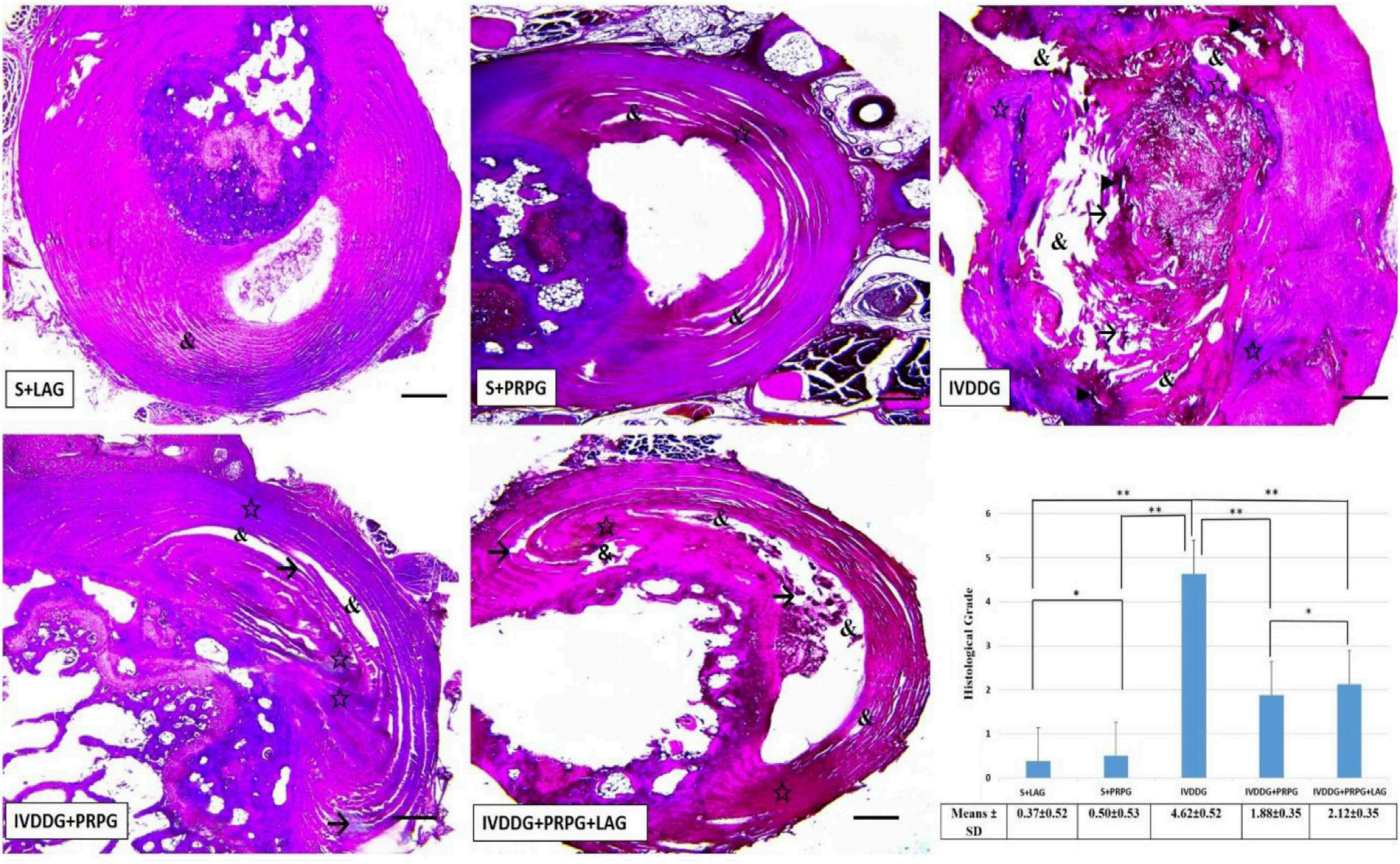
Representative light microscope images of Verhoeff-Van Gieson-stained intervertebral disc tissue sections. The S + LAG and S + PRPG groups showed normal intervertebral disc tissues. In the IVDDG group, vacuoles, tissue integrity deterioration, interstitial edema, necrotic tissue, and degeneration were observed. In the treatment groups, IVDDG + PRPG and IVDDG + PRPG + LAG, a small amount of edema, vacuoles, and tissue integrity deterioration were detected in the intervertebral disc tissue. Markings include: (&) vacuoles, (→) tissue integrity deterioration, (☆) interstitial edema, and (►) necrotic tissue. Low magnification (×4, scale bar = 200 µm). (a) The histological grades of different groups are presented as mean ± SD. **p < 0.05 statistically significant and *p > 0.05 not statistically significant.

In VVG staining assessing collagen fibers, the sham groups appeared normal. In IVDDG tissues, the boundaries between the AF and NP were indistinct, collagen fibers were disorganized and degenerated, and necrotic tissues, vacuoles, and interstitial edema were observed. In the treatment groups (IVDDG + PRPG and IVDDG + PRPG + LAG), atrophic structures disappeared, with reductions in vacuolization and edema, along with occasional degenerations in collagen fibers (Figure 3).

No significant differences were found between the S + LAG and S + PRPG groups in histological classifications based on VVG staining (p = 0.685). However, significant statistical differences were observed when comparing the sham groups (S + LAG and S + PRPG) with the IVDDG group (p < 0.001). Although statistical differences were noted between the IVDDG and the IVDDG + PRPG (p < 0.001) and IVDDG + PRPG + LAG (p < 0.001) groups, the average values of pathological findings decreased, indicating a regression of degeneration. A statistically significant reduction was found when comparing the IVDDG group with the IVDDG + PRPG and IVDDG + PRPG + LAG groups (p < 0.001) (Figure 3).

## Discussion

Platelet-rich plasma exerts a triggering effect on inflammation and tissue repair in damaged tissues due to its rich content of mediators, including transforming growth factor-β1 (TGF-β1), bone morphogenetic proteins 2 and 7 (BMP-2, BMP-7), insulin-like growth factor 1 (IGF-1), fibroblast growth factors 2 and 18 (FGF-2, FGF-18), vascular endothelial growth factor (VEGF), epidermal growth factor (EGF), and platelet-derived growth factor (PDGF). Thanks to these potent reparative mediators, PRP has found widespread applications in various fields. In orthopedics, it is commonly used to treat chronic conditions such as cartilage degeneration, disc degeneration, tendinitis, calcaneal spur, and lateral epicondylitis, as well as traumatic muscle, tendon, and ligament injuries and nonunion fractures (Mascarenhas et al., 2015; Fortier et al., 2011). Fortier et al. demonstrated in their animal study that PRP showed a regenerative effect on localized cartilage injuries and osteoarthritic cartilage both *in vivo* and *in vitro* (Fortier et al., 2011). Similarly, Patel et al. conducted a double-blind study on 78 patients with moderate to advanced osteoarthritis in their knees, comparing one group receiving PRP and another receiving saline injections, and found significantly higher Western Ontario and McMaster Universities Arthritis (WOMAC) scores in the PRP group (Patel et al., 2013).

A review of the literature reveals that many *in vitro* studies have demonstrated that PRP triggers excessive proliferation in adult mesenchymal cells and fibroblasts (Liu et al., 2002; Lucarelli et al., 2005). In alignment with these findings, Histological assessment of sections from the PRP-treated IVDDG + PRPG and IVDDG + PRPG + LAG groups revealed a significant reduction in necrotic tissue and vacuolization, accompanied by a marked increase in fibroblast proliferation and mesenchymal cell population (p < 0.05). This pattern was consistently observed across all histopathological sections.

Furthermore, PRP decreased atrophy and cellular clustering in NP cells, thus demonstrating its histological healing effects.

To support the histological evidence of healing, we assessed the biochemical parameters by measuring serum levels of IL-6 and TNF-α in samples collected after the rats were sacrificed. Comparisons of blood samples from the S + LAG and IVDDG groups revealed significantly higher levels of IL-6 and TNF-α in the IVDDG group. The sustained elevation of IL-6 and TNF-α levels in the IVDDG group at week five indicates that the healing process for disc degeneration is insufficient and ongoing. While there was no significant difference in TNF-α levels between the IVDDG and S + LAG groups, the statistically significant increase in IL-6 levels in the S + LAG group is considered a result of the injection effect. Comparisons between the IVDDG group and the S + PRP, IVDDG + PRPG, and IVDDG + PRPG + LAG groups showed statistically significant lower levels of IL-6 and TNF-α across all treatment groups, supporting PRP’s contribution to the histologically demonstrated healing in disc degeneration through biochemical parameters.

Although there was no statistically significant difference in IL-6 and TNF-α levels between the IVDDG + PRPG and IVDDG + PRPG + LAG groups, both treatment groups demonstrated significantly lower pro-inflammatory cytokine levels compared to the control groups that did not receive PRP or PRP combined with local anesthetic. This biochemical outcome supports the anti-inflammatory efficacy of PRP in intervertebral disc degeneration and suggests that the addition of a local anesthetic does not compromise the therapeutic potential of PRP. These findings align with the hypothesis that local anesthetics, when co-administered with PRP, do not interfere with its biological activity, at least at the cytokine level.

One of the primary aims of our study was to elucidate the healing effects of PRP, while another main objective was to determine whether the efficacy of PRP diminishes when combined with local anesthetics. A review of the literature revealed a lack of sufficient scientific data regarding whether PRP loses its effectiveness when combined with local anesthetics due to the painful nature of PRP application during and after the procedure. In an *in vitro* study by Dregalla et al., the authors evaluated the effects of bupivacaine, ropivacaine, and lidocaine mixed with PRP derived from fresh blood, assessing various parameters, including platelet morphology, viability, adhesion capacity, intracellular calcium levels, production of oxygen radicals, and apoptosis. Their findings indicated that ropivacaine and lidocaine did not have any adverse effects on the aforementioned parameters, while bupivacaine was claimed to negatively affect them (Dregalla et al., 2021). In our study, we aimed to evaluate the effects of ropivacaine mixed with PRP *in vivo* using experimental animal models, assessing both histological and biochemical parameters. The similar histological and biochemical results observed between the groups receiving only PRP and those receiving PRP combined with a local anesthetic suggest that ropivacaine does not exert any negative effects on PRP.

Numerous scientific studies have demonstrated that the primary effects of PRP are attributed to the growth factors contained within the platelets (Dregalla et al., 2021; Marx et al., 1998; Browner et al., 2014; Sanchez et al., 2003). Our study, which investigated the healing effects of PRP and the impact of local anesthetics on PRP *in vivo* using rat models of disc degeneration, found no statistically significant differences in serum IL-6 and TNF-α levels between treatment groups receiving PRP with local anesthetics and those without. This indicates that ropivacaine, at the studied dose, does not negatively affect the efficacy of PRP. Additionally, histological evaluations showed no significant differences between the treatment groups that used local anesthetics and those that did not, further supporting that ropivacaine does not adversely affect PRP.

A limitation of our study was the lack of evaluation of additional local anesthetic agents. Given the number of rats available under current conditions, we aimed to use them most efficiently to explore the effects of PRP on disc degeneration and the influence of local anesthetics on PRP. Future studies are warranted to further explore the mechanisms underlying PRP’s regenerative properties and the implications of combining it with various anesthetics in different patient populations.

In conclusion, this study confirmed the histological and biochemical healing effects of PRP in intervertebral disc degeneration and demonstrated that its efficacy was not diminished when combined with ropivacaine. Similar levels of serum IL-6 and TNF-α, along with histological findings, support that the use of local anesthetics does not adversely affect the anti-inflammatory and regenerative properties of PRP. These results suggest that local anesthetics can be safely used to improve patient comfort during PRP applications. However, further studies investigating the effects of different local anesthetics are warranted to fully understand the therapeutic potential of PRP.

## Data Availability

The original contributions presented in the study are included in the article/supplementary material, further inquiries can be directed to the corresponding author.
